# Introgression of Root and Water Use Efficiency Traits Enhances Water Productivity: An Evidence for Physiological Breeding in Rice (*Oryza sativa* L.)

**DOI:** 10.1186/s12284-019-0268-z

**Published:** 2019-03-07

**Authors:** Prathibha M. Dharmappa, Pushpa Doddaraju, Mohankumar V. Malagondanahalli, Raju B. Rangappa, N. M. Mallikarjuna, Sowmya H. Rajendrareddy, Ramachandra Ramanjinappa, Rajanna P. Mavinahalli, Trichy Ganesh Prasad, Makarla Udayakumar, Sreeman M. Sheshshayee

**Affiliations:** 1 Department of Crop Physiology, University of Agricultural Sciences, GKVK, Bengaluru, 560065 India; 2 Present address- Department of Agronomy Kansas State University, Kansas, USA; 3 Present address: Assistant Professor, Department of Biotechnology, Reva University, Bengaluru, India; 4 Rice Breeder, Zonal Agricultural Research Station, VC Farm, Mandya, 571405 India

**Keywords:** Aerobic Rice, Roots, Water-use efficiency, Quantitative trait loci, Marker assisted backcross breeding, Carbon isotope discrimination, Phenotyping, Trait introgressed lines, Grain yield

## Abstract

**Background:**

Semi-irrigated aerobic cultivation of rice has been suggested as a potential water saving agronomy. However, suitable cultivars are needed in order to sustain yield levels. An introgression of water mining and water use efficiency (WUE) traits is the most appropriate strategy for a comprehensive genetic enhancement to develop such rice cultivars.

**Results:**

We report a novel strategy of phenotyping and marker-assisted backcross breeding to introgress water mining (root) and water use efficiency (WUE) traits into a popular high yielding cultivar, IR-64. Trait donor genotypes for root (AC-39020) and WUE (IET-16348) were crossed separately and the resultant F_1_s were inter-mated to generate double cross F_1_s (DCF_1_). Progenies of three generations of backcross followed by selfing were charatcerised for target phenotype and genome integration. A set of 260 trait introgressed lines were identified. Root weight and root length of TILs were 53% and 23.5% higher, while Δ^13^C was 2.85‰ lower indicating a significant increase in WUE over IR-64. Five best TILs selected from BC_3_F_3_ generation showed 52% and 63% increase in yield over IR-64 under 100% and 60% FC, respectively. The trait introgressed lines resembled IR64 with more than 97% of genome recovered with a significant yield advantage under semi-irrigated aerobic conditions The study validated markers identified earlier by association mapping.

**Conclusion:**

Introgression of root and WUE into IR64, resulted in an excellent yield advantage even when cultivated under semi-irrigated aerobic condition. The study provided a proof-of-concept that maintaining leaf turgor and carbon metabolism results in improved adaptation to water limited conditions and sustains productivity. A marker based multi-parent backcross breeding is an appropriate approach for trait introgression. The trait introgressed lines developed can be effectively used in future crop improvement programs as donor lines for both root and WUE.

**Electronic supplementary material:**

The online version of this article (10.1186/s12284-019-0268-z) contains supplementary material, which is available to authorized users.

## Background

Rice, that meets over half of the global dietary requirement and 80% of Asians, is an exhaustive user of fresh water. With increasing water scarcity, cultivating rice in the conventional puddled ecosystem is increasingly becoming uneconomical (Bouman et al. 2005, 2007; Nie et al. 2012). Several agronomic practices such as direct seeding, alternate wetting and drying, and aerobic cultivation have been suggested as water-saving practices (Bouman and Tuong 2001; Atlin et al. 2006; Bouman et al. 2007). The semi-irrigated aerobic cultivation though can potentially save up to 50% water, a yield penalty of 40% to 50% is often reported (De-Datta and Feuer 1975; Widawsky and O’Toole 1990; Fischer et al. 2003; Bouman et al. 2005; Peng et al. 2006; Kreye et al. 2009; Sasaki et al. 2010). This approach involves periodic surface irrigation to bring the soil to 100% field capacity (FC)) (Bouman and Tuong 2001) which periodically results in the crop experiencing soil moisture stress between episodes of irrigation. Higher VPDs, characteristic to semi-irrigated aerobic environments further reduce carbon assimilation through partial stomatal closure (Carmelita et al. 2011; Turc et al. 2011; Lobell and Gourdji 2012). Periodic soil moisture depletion exacerbates yield loss due to increased spikelet sterility besides decreasing carbon assimilatory capacity. Therefore, it is imperative that suitable genetic enhancement of rice crop must be achieved to harness the water saving advantages of semi-irrigated aerobic cultivation.

Breeding effort to sustain productivity was predominantly achieved through selection for absolute yield under stress (Affholder et al. 2013; Kumar et al. 2014; Dixit et al. 2014). A narrow genetic variability in yield among the high yielding cultivars and a large G x E interaction for yield severely limit further progress in improving yields (Araus et al. 2008; Reynolds and Tuberosa 2008). Thus, a focused trait-based breeding is strongly being professed for a more effective genetic enhancement in rice productivity under aerobic condition (Reynolds and Tuberosa 2008; Reynolds and Langridge 2016; Sheshshayee et al. 2018).

There have been significant progress in recent years in enumerating and deciphering component traits that are essential for improving drought adaptation (Serraj et al. 2011; Vadez et al. 2013; Araus and Cairns 2014). Based on large number of systematic investigations, it is evident that maintenance of tissue turgor through better water uptake, conservation and maintenance of carbon assimilatory capacity even under conditions of decreasing turgor have the greatest relevance for drought adaptation (Blum 2011; Lopes et al. 2011; Bartlett et al. 2012; Osakabe et al. 2014; Meinzer et al. 2014; Sheshshayee et al. 2018).For a comprehensive improvement of drought adaptation, it is desirable that these constitutive traits are introgressed (Blum 2011; Tardieu 2012) onto an elite genetic background.

While the relevance of root traits in water uptake is unequivocally accepted (Gowda et al. 2011; Henry et al. 2011; Kitomi et al. 2015; Price and Tomos 1997; Serraj et al. 2011; Steel et al. 2006, 2013; Uga et al. 2011, 2012, 2013, 2015; White et al. 2015), the relevance of water use efficiency has been debated (Blum 2011; Sheshshayee et al. 2012). We provided convincing experimental evidences demonstrating the relevance of WUE in improving drought adaptation (Sheshshayee et al. 2012). Similar to the observations made by Reynolds and Langridge (2016), we also provided evidences that the greatest impact on drought adaptation is noticed only when relevant traits are introgressed on to a single genetic background (Raju et al. 2014).

Recently, through association genetic approach we identified a few robust markers governing WUE, root and other physiological traits along with specific trait donor genotypes (Raju et al. 2016). IR64, one of the popular Mega varieties cultivated under puddle conditions, is highly sensitive to water deficit conditions. The major goal of the present investigation was to introgress root and WUE traits into the genetic background of IR-64. A novel approach for introgressing specific traits through marker assisted backcross breeding combined with phenotypic selection was deployed.

We demonstrate that the trait introgressed lines exhibit superior productivity even under water limiting semi-irrigated aerobic conditions. To the best of our knowledge, this is the first successful effort for introgressing complex physiological traits for improving drought adaptation in rice, an approach gaining prominence as physiological breeding.

## Methods

### Development of Backcross Progenies

Extensive phenotypic and molecular characterization of 173 *Indica* rice (*Oryza sativa* L.) germplasm for drought adaptive traits led to identification of trait specific SSR markers and trait donor lines, AC-39020 and IET-16348 for root and WUE, respectively (Raju et al. 2016). IR-64, a lowland high yielding rice variety was selected as a recurrent parent to introgress root and WUE traits through marker assisted backcross breeding.

### Breeding Strategy

#### Marker Analysis at Early Backcross Generations

Markers associated with root and WUE identified from association mapping were used in the study. Sixteen markers (12 for root traits and 4 for WUE) associated with traits and 120 non-target markers were used for foreground and background selection, respectively. Details of the SSR markers used for foreground selection and background selection are given in Table 1 and Additional file 1: Table S1.

**Table 1:** List of markers associated with specific target trait used for Foreground selection

Trait associated	Trait component	Marker	Chr. no	Position on chromosome (cM)	R^2^	IR-64	AC-39020	IET-16348
Root	RLD	RM80	8	103.7	19.0	132	143	122
	RWT	RM2584	8	45.8	14.5	173	176	247
	RLD	RM1388	4	77.9	17.2	245	235	215
	RLD	RM262	2	81.1	14.5	185	178	170
	R/S	RM239	10	25.2	13.8	194	198	219
	RV	RM3825	1	143.7	13.0	163	169	154
	RV	RM16	3	131.5	10.8	186	187	167
	RL	RM3276	4	102.4	13.3	158	190	169
	RV	RM247	12	32.3	12.1	145	139	166
	RLD	RM167	4	37.5	16.7	167	129	146
	RV	RM4455	10	21.8	20.4	221	298	149
	R/S	RM71	2	49.8	10.1	160	143	168
Δ^13^C	Δ^13^C	RM493	1	79.9	14.2	260	250	240
	Δ^13^C	RM586	6	7.4	17.4	243	272	278
	Δ^13^C	RM149	8	122.1	15.4	255	264	357
	Δ^13^C	RM131	4	148.8	18.3	217	207	223

Note: These markers were discovered by adopting association mapping approach and are reported in Raju et al. (2016). The position of the marker on rice genome was obtained from http://archive.gramene.org/markers/

*RLD* Root length density (cm^2^ g^− 1^), *RWT* Root weight (g pl^− 1^), *R/S* Root to shoot biomass ratio (g. g^− 1^), *RV* Root volume (cm^3^), *Δ*^*13*^*C* Carbon isotope discrimination (‰)

#### Phenotyping for WUE and Roots at Early Backcross Generations

Besides molecular characterization of progeny, specific proxies for the root and WUE were also measured at each backcross generation. Carbon isotope discrimination (Δ^13^C), a well-known proxy for WUE (Farquhar et al. 1989) was measured to assess the differences in WUE. Similarly, a non-invasive approach of measuring leaf temperature using infrared thermometer (SCHEDULER Plant Stress-Monitor, USA) was adopted as an indirect estimate of transpiration and hence root traits at early generations of backcrossing (Anda and Ligetvári 1993). At advanced generations after attaining homozyosity, root traits were measured by growing plants in root structures (see later).

**Fig. 1:**
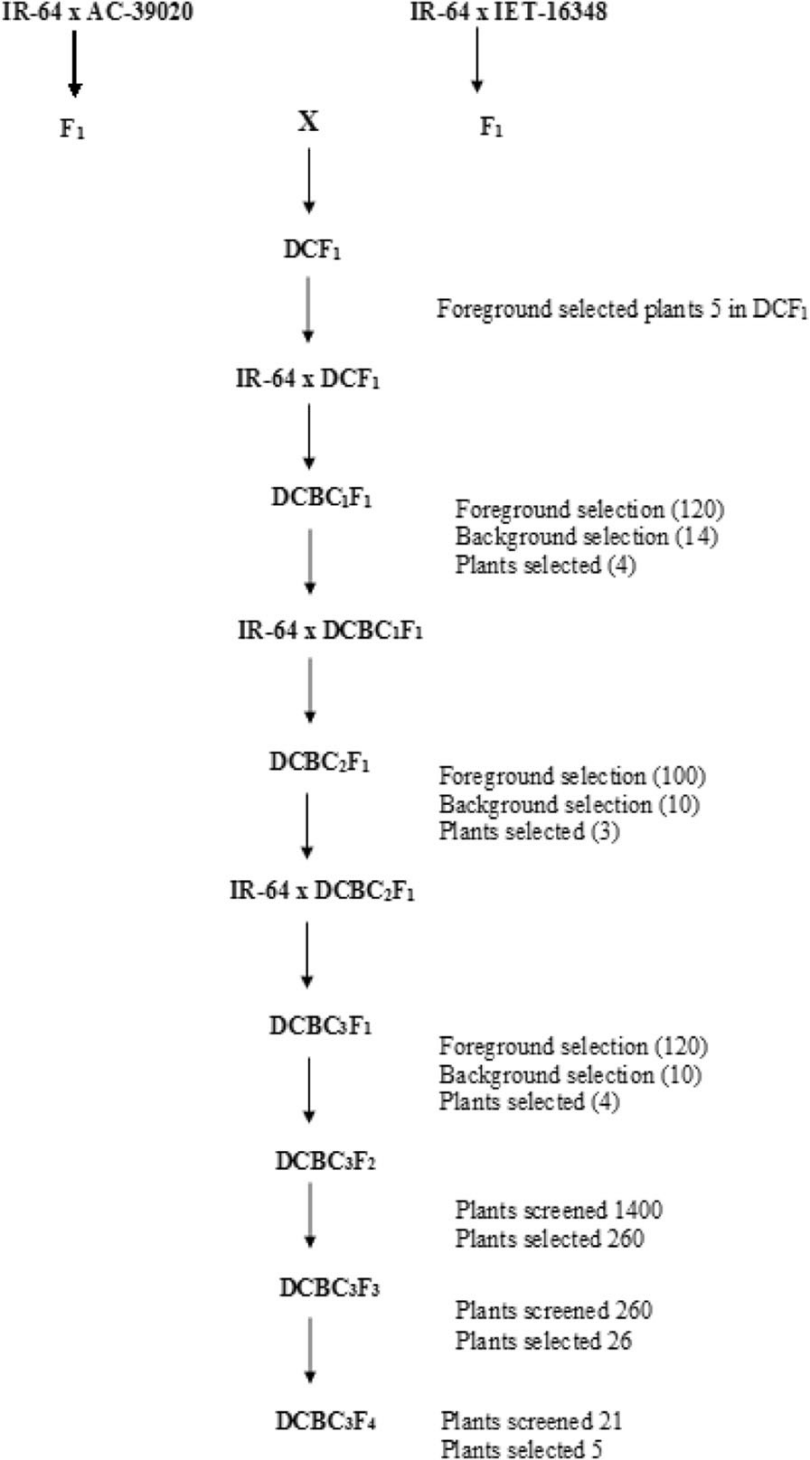
The scheme of Multi-parent Marker-assisted backcrossing (MABC) to introgress root and WUE traits into IR-64 background. Foot note: Based on phenotypic and molecular diversity AC-39020 and IET-16348 were selected as donor parents for root WUE associated traits. IR-64 was selected as recipient parent because of its drought susceptibility. The donor parents were hybridised with recipient parents separately, to obtain the F_1_s. The resultant F_1_s were hybridised to get DCF_1_s. From this stage, plants were selected and backcrossed with IR-64 till BC_3_F_1_ stage. Further, selfing was done to identify the trait introgressed lines

Two crosses were effected separately to introgress root and WUE traits into IR64 (Fig. 1). True F_1_ plants were identified using SSR markers associated with target traits (Table 1). The identified true F_1_s were inter-mated to develop double-cross F_1_s (DCF_1_).

At each generation, molecular and phenotypic characterization was performed to identify best trait introgreesed lines with maximum number of target marker loci in heterozygous condition and trait expression using proxies. These best trait introgressed line were backcrossed with IR-64 to generate backcross progenies till BC_3_ generation. See Fig. 1 for details.

Four best BC_3_F_1_ plants (foreground selection for the target markers, background selection for maximum recurrent parent genome recovery and the proxy phenotype traits) were selfed to generate BC_3_F_2_ seeds (more than 10,000). A set of 1440 BC_3_F_2_ plants were taken for phenotyping growth and yield parameters under aerobic field condition. A set of selected 260 promising lines were advanced and the BC_3_F_3_ lines were phenotyped for root and WUE traits by raising the plants in specially designed root structures (Additional file 1: Figure S1) (Raju et al. 2014, 2016). The same set of 260 BC_3_F_3_ lines were phenotyped for yield and yield attributes under aerobic field condition. Based on field experiments, Five BC_3_F_4_ lines were selected for assessing the yield performance under aerobic field conditions using a managed drought Environment (MDE) facility.

#### PCR Conditions for Genotyping

DNA was extracted from young leaves of two-week-old plants using modified CTAB method (Saghai-Maroof et al. 1984). The DNA fragments were amplified using 30 ng template DNA, 1.5 μL PCR buffer (10X), 1.5 μL dNTPs (2 mM), 1.5 μL forward and reverse primer (5 pmol/μL), 1 U *Taq* DNA polymerase and volume made up to 15 μL with sterile water. All the amplified products were analysed on microchip based electrophoresis system MultiNA (Shimadzu biotech, Japan) (Mathithumilan et al. 2013).

#### Description of Phenotyping Approaches

The set of 260 most promising trait introgressed BC_3_F_3_ lines were used for phenotyping water mining traits (roots), yield and yield attributes under semi-irrigated aerobic conditions.

#### Stable Carbon Isotope Discrimination (Δ^13^C)

Stable carbon isotope composition (δ^13^Cp) was measured using an isotope ratio mass spectrometer (DeltaV Adv. Thermo Fisher Scientific, Bremen, Germany) interfaced with an elemental analyzer (NA1112, Carlo Erba, Italy) through a continuous flow device (ConFlo III, Thermo Fisher Scientific) installed in the Department of Crop Physiology, UAS, Bangalore, as a national facility. At active tillering stage (45–50 DAS), the second fully expanded leaves were collected from all germplasm separately and dried. The dried leaf samples were homogenized to a fine powder with a ball mill. Three replications from each genotype were used for the measurement of isotope ratios. Carbon isotope discrimination Δ^13^C, expressed in per mill (‰), was computed considering the isotopic composition of air (δ^13^C_a_) as − 8‰ relative to Vienna Pee Dee Belemnite (VPDB), as follows (Farquhar et al. 1989):

$$ {\Delta}^{13}\mathrm{C}=\frac{\left({\updelta}^{13}{\mathrm{C}}_{\mathrm{a}}\hbox{-} {\updelta}^{13}{\mathrm{C}}_{\mathrm{p}}\right)}{\left(1+{\updelta}^{13}\mathrm{C}\mathrm{p}/1000\right)} $$

The analytical uncertainty of measurement was determined using a laboratory standard (Potato Starch (C_3_), Sigma-Aldrich, δ^13^C = − 26.85‰) and found to be better than 0.15‰. The laboratory standard was calibrated against international standards like ANU Sucrose.

#### Leaf Temperature as a Proxy for Root Traits in Early Generations

The leaf temperature of backcross progenies was measured using an infra-red gun (SCHEDULER Plant Stress-Monitor, USA). The gun was held pointing towards the leaf surface at 25 cm and the measurements were made at mid-day when there was bright sunlight.

#### Phenotyping Root Traits in Advanced Generations

Phenotyping root traits was carried out at the Department of Crop physiology, GKVK, campus of University of Agricultural Sciences, Bengaluru, India located at 12°58′N, 77° 35′E, 930 m above mean sea level. Weather data for the GKVK campus during the experimental period are given in Additional file 1: Table S2. Plants were grown in root structures, measuring 150 cm tall, 300 cm wide and 1800 cm long, built using cement bricks. An additional 150-cm-tall wall was built in the middle of the structure all along the length to make two halves, each 150 cm wide (Sheshshayee et al. 2011; Raju et al. 2014, 2016). The root structures were filled with red sandy loam soil m1ixed with FYM in 3:1 proportion and compacted to mimic the real field condition. The GKVK campus soil typically holds 22–24% water (*W*/W) at 100% field capacity (FC) with a pH of 6.5 and bulk density of 1.5 mg cm^− 3^ (Sunil and Shankaralingappa 2014). Twenty-one-day-old seedlings were transplanted in a randomized block design (RBD) with three replications. Each replication comprised of one row with 8 plants at a spacing of 25 cm between plants and 25 cm between rows. The advantage of this approach is the maintenance of the same plant population as that in the field, thus enabling a realistic phenotypic expression. Standard recommendations of fertilizers (100:50:50 kg NPK/ha) and other prophylactic measures were taken to raise a healthy crop. The soil moisture status was constantly maintained between 85 and 95% field capacity (to mimic the real field aerobic situation). Calibrated soil moisture probes (Gopher 9.2, Dataflow Systems Pvt. Ltd., Christchurch, New Zealand) were inserted at various places in the root structures to determine the moisture content at different soil depth and irrigation was scheduled based on the soil moisture data. This method of scheduling irrigation based on soil moisture data ensured maintenance of soil moisture status at a given level despite rains during the experiment. Adequate drainage holes were provided at regular intervals on the walls of the root structure to ensure no stagnation of water. On the 75th day after sowing (DAS), the sidewalls of the root structures were dismantled and roots were extracted carefully with a jet of water to wash away the soil from roots. At the time of harvest, roots were separated from the shoot and several parameters such as root length, root volume (amount of water displaced by fresh roots) and total canopy leaf area were recorded before drying the samples in a hot air oven at 75 °C until constant weights were reached for recording dry biomass. Leaves were carefully separated from the shoot and roots were cut off and oven dried separately.

#### Phenotyping Yield Under Aerobic Condition

Seedling (22 days old) of the selected 260 lines were transplanted in the main field with a spacing of 25 × 25 cm to evaluate their performance under aerobic condition. Surface irrigation was scheduled once in 5 days. At maturity, the entire plant was cut 1 inch above the ground level and the panicles were separated from the stem. The panicles were sundried, whereas the stem samples along with the leaves were oven dried at 75 °C until constant weights were attained.

#### Phenotyping Under Managed Drought Environment (MDE) to Evaluate Stress Response

Five most promising lines from among the 260 TILs were selected for this study. The selected Five TILs along with parental lines were grown under this MDE facility with the spacing of 25 × 25 cm with three replications. Two soil moisture regimes viz., well-watered (100% FC) and water-deficit (60% FC) were maintained by sprinkling 10 mm of water on alternate days for well-watered and once in three to 5 days for the water-stressed plots for the entire crop growth period. Sprinkler irrigation schedules were determined based on the soil moisture status measured using soil moisture probes (Gopher 9.2, Dataflow Systems Pty Ltd., Christchurch, New Zealand) inserted at several places in the plots of each water regime treatment. Leaf samples were collected at 50 DAS for measuring relative water content (RWC), ∆^13^C specific leaf area (SLA). At final harvest, parameters such as spikelet fertility, grain yield and total above ground biomass were measured.

### Relative Water Content (RWC)

Pieces of a known area of fresh leaves were taken from the first fully expanded leaf from the apex of the plant at 9.00 am and its fresh weight was determined immediately. The leaf bits were immersed in water taken in a beaker and kept for a period of at least 6 h in dark. The leaf bits were carefully blotted to remove water on the leaf surface and weighed accurately to determine the turgid weight. The leaf bits were then oven dried and the dry weight was recorded. Relative water content was computed as follows (Morgan 1995),

$$ \mathrm{RWC}=\frac{\left(\mathrm{Fw}\hbox{-} \mathrm{Dw}\right)}{\left(\mathrm{Tw}\hbox{-} \mathrm{Dw}\right)}\times 100 $$

where: Fw = Fresh weight; Tw = Turgid weight; and Dw = Dry weight

### Specific Leaf Area (SLA)

Also referred to as leaf area to mass ratio, is a reflection of leaf spread and hence the surface area for transpiration and photosynthesis. Leaf samples were collected from the second fully expanded leaf from the apex of the plant. Middle 15 cm long leaf lamina was cut and the average width was determined. The leaf samples were oven dried at 75 °C and dry weights were recorded after constant weights were attained. SLA was computed as follows (Wilson et al. 1999).

$$ \mathrm{SLA}=\frac{\mathrm{Leaf}\kern0.5em \mathrm{area}}{\mathrm{Leaf}\kern0.5em \mathrm{weight}} $$

### Statistical Analysis

Analysis of Variance was calculated using SPSSv16.0 version. F-test and student’s t-test was performed to identify the significant difference among the backcross progenies. Critical Difference (CD) at *P* ≤ 0.05 was used to calculate the treatment means. Error bars were used to represent standard error among TILs in all figures. Graphical genotyping of the TILs was performed using the software GGT 2.0.

## Results and Discussion

Saving irrigation water and sustaining productivity that have emerged as major research goals can be achived by improving effective extraction of water and efficient use of water for biomass production. Demonstrating that root system is an important trait, notwithstanding, the relevance of water use efficiency (WUE) in improving growth rates has been debated (Blum 2011). We demonstrated that WUE significantly contributes to growth rates in genotypes that used comparable volumes of water (Passioura 2006; Tuberosa 2012; Sheshshayee et al. 2012; Reynolds and Langridge 2016). For a comprehensive improvement in saving water and sustaining productivity, water mining (roots) and WUE need to be introgressed on to a single elite genetic background (Raju et al. 2014; Sheshshayee et al. 2018). Therefore, the major goal of this investigation was to introgress root and WUE traits into IR-64, a high yielding mega variety (Mackill and Khush 2018). We adopted a multi-parent marker assisted backcross breeding strategy to introgress these traits.

### Selection of Parents

While strongly linked DNA markers can accelerate selection of appropriate progeny, identification of robuts trait donor genotypes is equally important.

IR64, developed at the International Rice Research Institute (IRRI), Philippines, is one of the mega varieties of rice cultivated widely in India. The popularity of this variety was primarily due to its high yield potential, better cooking quality/taste and resistance to a few important biotic stresses like leaf blast, bacterial blight and brown plant hopper (Mackill and Khush 2018). But it is well documented that the yield of IR-64 reduces up to 50% under water limited conditions (Uga et al. 2013). Hence we selected IR-64 as the recurrent parent to intorgress drought-adaptive traits to improve its productivity under water limited conditions.

In an initial experiment, a panel of *Indica* rice germplasm were extensively characterized for phenotypic and molecular diversity to identify robustb QTL and trait donor genotypes (Raju et al. 2016). The two donor lines, viz. AC-39020 (for root) and IET-16348 (for WUE) were used for trait introgression through marker assisted backcross breeding into IR-64 background.

The profile of associated polymorphic markers and phenotype of these donor lines in comparison with the recurrent parent are given in Tables 1 and 2.

**Table 2:** Phenotypic variations and marker polymorphism of the trait donor genotypes in comparison with IR-64

Parent	RL (cm)	RW (g pl^−1^)	RV (cm^3^)	Δ^13^C (‰)	TLA (cm^2^ pl^−1^)	TDM (g pl^− 1^)	DM/LA (g m^2^)	GL/GW
IR-64	25.5	2.5	25.1	21.5	3543	65.61	120.76	2.11
AC-39020	65.4	10.4	70.3	19.5	4563	120.75	188.45	1.42
IET-16348	35.5	5.1	50.4	18.4	3278	150.58	150.28	1.81

Note: *RL* Root length, *RW* Root weight, *RV* Root volume, *Δ*^*13*^*C* Carbon isotope discrimination, *TLA* Total leaf area, *TDM* Total dry matter, *DM/LA* Dry matter per unit leaf area, *GL/GW* Grain length to width ratio

### Phenotyping for Target Traits Using Proxies

High throughput phenotyping for root traits and WUE has always been a challenge. Plants ability to discriminate against the heavy isotope of carbon during photosynthesis (Δ^13^C) has been established as a well-accepted surrogate for WUE on a time integrated scale (Farquhar et al. 1989). The relevance of Δ^13^C as a powerful surrogate has been validated in several C_3_ crop species including rice (Impa et al. 2005). While Δ^13^C is a fairly high throughput measurement option, phenotyping for root traits is still challenging. We developed a simple approach of growing plants in raised root structures specially constructed for root phenotyping. However, the disadvantage of this approach is the requirement of destructive sampling and hence will not be suitable for early generation plants.

Transpiration is well known for its role in evaporative cooling besides other advantages in carbon assimilation. At a given VPD, transpiration would be a function of water extraction and hence root traits (Vadez 2014; Reynolds et al. 2016). Hence, canopy temperature is a good proxy for transpiration and hence root traits. We determined the leaf temperatures as a proxy for root traits in early generations while actual root measurements were made in more stabilizing plants (after BC_3_F_3_).

### Trait Introgression and Selection of Introgressed Lines

IR64 was crossed separately with each of the trait donor genotypes and the resultant F_1_s were inter-mated to develop double cross F_1_ (DCF_1_) plants. True DCF_1_ plants that showed heterozygosity at each of the selected marker loci were identified and used for backcrossing with IR-64. Plants at each of the early backcross generations were phenotyped for Δ^13^C and leaf temperature and genotyped with SSR markers for foreground and background selections. The scheme of crossing and the number of plants selected by markers as well as phenotype for backcrossing is illustrated in Fig. 1.

### Phenotypic and Molecular Analysis of Backcross Progenies

Five true DCF_1_ plants were backcrossed with IR-64 used as the female recurrent parent. This cross resulted in generating 120 BC_1_F_1_ plants which were genotyped for foreground selection using associated SSR markers. A set of 42 BC_1_F_1_ plants with heterozygosity at most of the target loci were phenotyped for Δ^13^C and leaf temperature at grand growth stage. The mean and range for these traits are given in Table 3A. Based on the foreground marker profile and phenotype, 14 BC_1_F_1_ plants were characterised for recurrent parent genome recovery. This led to the identification of four BC_1_F_1_ plants which were selected for further backcrossing. The results of Δ^13^C and leaf temperature for these four selected lines are presented in Table 3B. The mean Δ^13^C of the four selected BC_1_F_1_ plants was 19.45‰ and mean leaf temperature was 33.05 °C. The cooler leaves and lower Δ^13^C of the selected introgressed lines compared with IR64 indicate increased WUE and root traits in the progeny.

**Table 3:** Morpho-physiological and molecular variations of IR-64 and the backcross progenies at different generations (A) mean and range for the phenotypic value of target traits (B) improvement in traits over IR-64 at each generations, number of markers introgressed and background genome recovery in advanced backcross stages

(A)											
Generation	Season	# of plants		Δ^13^C (‰)				LT (° C)			
				IR-64	Mean ± SD	Range		IR-64	Mean ± SD	Range	
BC_1_F_1_^a^	June–December,2012	42		21.5	21.23 ± 0.95	17.5–25.5		35.6	32.3 ± 2.04	32.4–36.4	
BC_2_F_1_	June–December,2014	100		21.88	19.7 ± 0.87	18–25.3		37.2	35.0 ± 1.87	31.4–36.52	
BC_3_F_1_	January–May, 2015	120		22.65	18.84 ± 0.93	15.09–20.62		36.3	34.34 ± 1.73	30.2–36.5	
(B)											
Generation	Plants selected	# of markers in heterozygote condition		Genome recovery (%)		Phenotype		% change over IR-64		Significance	
		Roots (12)	Δ^13^C (4)	Expected	Estimated	Δ^13^C	LT	Δ^13^C (−ve)	LT (−ve)	Δ^13^C	LT
BC_1_F_1_	32	12	4	75.0	72.5	19.5	32.1	9.30	9.83	**	*
	73	11	3	75.0	69.7	19.1	33.5	11.16	5.90		
	81	12	4	75.0	70.4	19.8	32.5	7.91	8.71		
	91	11	2	75.0	73.2	19.4	34.1	9.77	4.21		
BC_2_F_1_	32–1	12	4	87.5	83.2	18.28	31.6	16.45	15.05	**	**
	73–3	10	3	87.5	82.8	18.65	33.2	14.76	10.75		
	81–23	12	4	87.5	82.1	18.42	31.4	15.81	15.59		
BC_3_F_1_	32–1-34	11	3	93.75	90.5	19.96	32.1	11.88	11.57	**	**
	32–1-40	10	4	93.75	90.5	18.35	31.5	18.98	13.22		
	81–23-27	10	3	93.75	91.5	17.82	32.7	21.32	9.92		
	81–23-31	10	4	93.75	92.5	18.85	31.1	16.78	14.33		

*Δ*^*13*^*C* Carbon isotope discrimination (‰), *LT* Leaf temperature (°C)

^a^ Since this was an early generation only 42 BC_1_F_1_ plants were phenotyped out of the total 120 BC_1_F_1_ plants generated

* and ** denote significance at 5% and 1%, respectively

Backcrossing of these four selected BC_1_F_1_ plants with IR-64 generated 100 BC_2_F_1_ plants. Based on FGS and phenotyping, 10 plants with maximum number of foreground markers in heterozygous state with lower Δ^13^C and cooler canopy, were used for background selection. Upon background selection, three lines were further identified. These three lines showed an average of 82.7% background genome recovery. The theoretically expected background genome recovery at BC_2_ stage is 87.5% (Collard et al. 2005). The lower percentage of background genome recovery in our experiment was because of the use of two diverse donor parents for trait introgression. The average Δ^13^C of the selected three BC_2_F_1_ plants was 18.45‰ and the leaf temperature was 32.07 °C, which were significantly lower than IR64 (Table 3B). Generally, BC_2_F_1_ plants are selfed to achieve homozygosity of the target loci. This strategy works best when only one donor parent is crossed with the recurrent parent. Since the observed average genome recovery was only 82.7% in our case, the three most promising BC_2_F_1_ plants namely 32–1, 73–3 and 81–23 with maximum foreground markers were backcrossed with IR-64 to generate a total of 120 BC_3_F_1_ plants.

These 120 BC_3_F_1_ plants were again genotyped for target loci and phenotyped for Δ^13^C and leaf temperature. Based on foreground selection and phenotyping, 10 BC_3_F_1_ plants that displayed heterozygosity at ten or more marker loci were further selected for background screening. Four individual plants namely 32–1-34, 32–1-40, 81–23-27, 81–23-31 representing > 90% of background genome recovery of IR-64 were selected. The mean Δ^13^C of the selected BC_3_F_1_ plants was 18.75‰ and leaf temperature of 31.85 °C (Table 3B). Field performance in terms of grain yield was possible to be recorded only from BC_3_F_1_ generation onwards. The yield of the four selected BC_3_F_1_ plants was generally higher than IR-64 (Additional file 1: Table S3) convincingly demonstrating the importance of introgressing WUE and root traits onto the same genetic background.

We observed that the background genome recovery did not increase as expected as backcrossing advanced. This could have been because of the less number of true double cross F_1_s selected at the earlier stage. However, a combination of molecular analysis with phenotypic selection would ensure that the minor alleles would not be missed. The markers used for target traits were discovered by association mapping and hence would be single markers that do not represent a genetic interval. Phenotyping for the target traits would be extremely essential to avoid the loss of minor allelic effects while selecting the progeny without interval markers.

The novelty of this approach was the simultaneous screening of the progeny for marker integration and phenotypic improvement. This strategy of backcross breeding provided a very strong validation for the markers identified by association mapping besides accelerating development of TILs in the elite genetic background of IR-64.

The selected BC_3_F_1_ plants were selfed to generate a large repository of BC_3_F_2_ seeds. The rationale of the advancement was that we identified four best BC_3_F_1_ plants with maximum number of foreground markers, highest yield and highest background genome recovery and picked 500 BC_3_F_2_ seeds from each of the selected BC_3_F_1_ plants for further characterization under semi-irrigated field conditions.

### Molecular and Phenotypic Characterization of BC_3_F_2_

Markers in LD with the target traits were used for screening a set of 1440 segregating progenies at BC_3_F_2_ generation. A presentative gel image derived from MultiNA is given in Additional file 3: Figure S4A. At this stage the selection was performed for homozygosity at each of the target marker locus.

### Field Evaluation of BC_3_F_2_ Plants

A set of 1440 BC_3_F_2_ plants were selected and raised in field under semi-irrigated aerobic condition. Specific leaf area (SLA) was determined as yet another proxy for WUE (Sheshshayee et al. 2006). SLA was lowest in IET-16348, indicating high WUE while it was high for IR-64 (Table 4). The average SLA for the 1440 BC_3_F_2_ plants was 170 cm^2^ g^− 1^ which was lower than that of the trait donor parent, IET-16348. The SLA ranged between 111 cm^2^ g^− 1^ and 287 cm^2^ g^− 1^, skewed towards the trait donor parent type indicating a targeted improvement in WUE. Grain yield and biomass also revealed a skewed distribution, though the traits varied between 4 and 55 g pl^− 1^ and 12–147 g pl^− 1^, respectively. The mean grain yield of the BC_3_F_2_ progenies was significantly higher than IR-64 (Additional file 1: Figure S2). Though favourable alleles of root and WUE were introgressed, there is a possibility where combination of unfavourable alleles may occur. The frequency distribution of grain yield, TDM along with other traits illustrated in Additional file 1: Figure S3.

**Table 4:** Performance of parents and the segregating BC_3_F_2_ (*n* = 1440) progenies for various morpho-physiological traits under semi-irrigated aerobic condition

Parents/Progenies	SLA	GY	TDM	DM/LA
IR-64	210.8 ± 16.5	20.3 ± 3.2	70 ± 6.3	116.4
AC-39020	196.28 ± 21.2	16.5 ± 5.4	103 ± 8.5	183.3
IET-16348	180.9 ± 14.3	14.6 ± 3.5	81.5 ± 5.4	160.5
Mean of1440 BC_3_F_2_ plants	170.46	35.50	74.97	145.3
Range	(111–287)	(4–55.5)	(12–147)	(100–189)
Selected 260 plants	204.5	44.06	103.5	154.6
CD	81.3	4.5	8.6	9.3
CV	12.4	8.6	12.3	13.4

Note: *SLA* Specific leaf area (cm^2^ g^−1^), *GY* Grain yield (g pl^−1^), *TDM* Total dry matter (g pl^−1^), *DM/LA* Dry matter per unit leaf area (g m^−2^)

We suggest that when water acquisition and water use efficiency traits are introgressed, plants can effectively use water for growth. Increase in WUE without a reduction in transpiration is only possible when a genotype has higher photosynthetic capacity (Udayakumar et al. 1998; Sheshshayee et al. 2003, 2012, 2018). A significant increase in DM/LA, an indicator of net assimilation rate among the backcross progenies (Table 4) illustrates the improved carbon assimilation capacity among the TILs. To further verify the performance of TILs, a set of 260 BC_3_F_2_ lines were selected based on grain yield and TDM. The mean and range for grain yield and TDM of these selected 260 BC_3_F_3_ lines are given in Table 4.

### Molecular and Phenotypic Characterization of BC_3_F_3_ Lines

The marker profiles of all the 260 lines are given in Additional file 2: Table S4. In advancedbackcross progeny (BC_3_F_3_), homozygosity of the target loci were screened to confirm the donor alleles (Additional file 3: Figure S4B).

To examine the trait introgression and its relevance, two elaborate phenotypic experiments were carried out. Root phenotyping was done in root structure while yield was monitored under aerocbic field conditions in two separate experiments.

### Experiment 1: Phenotyping for Root and WUE Traits of BC_3_F_3_ Lines

**Fig. 2:**
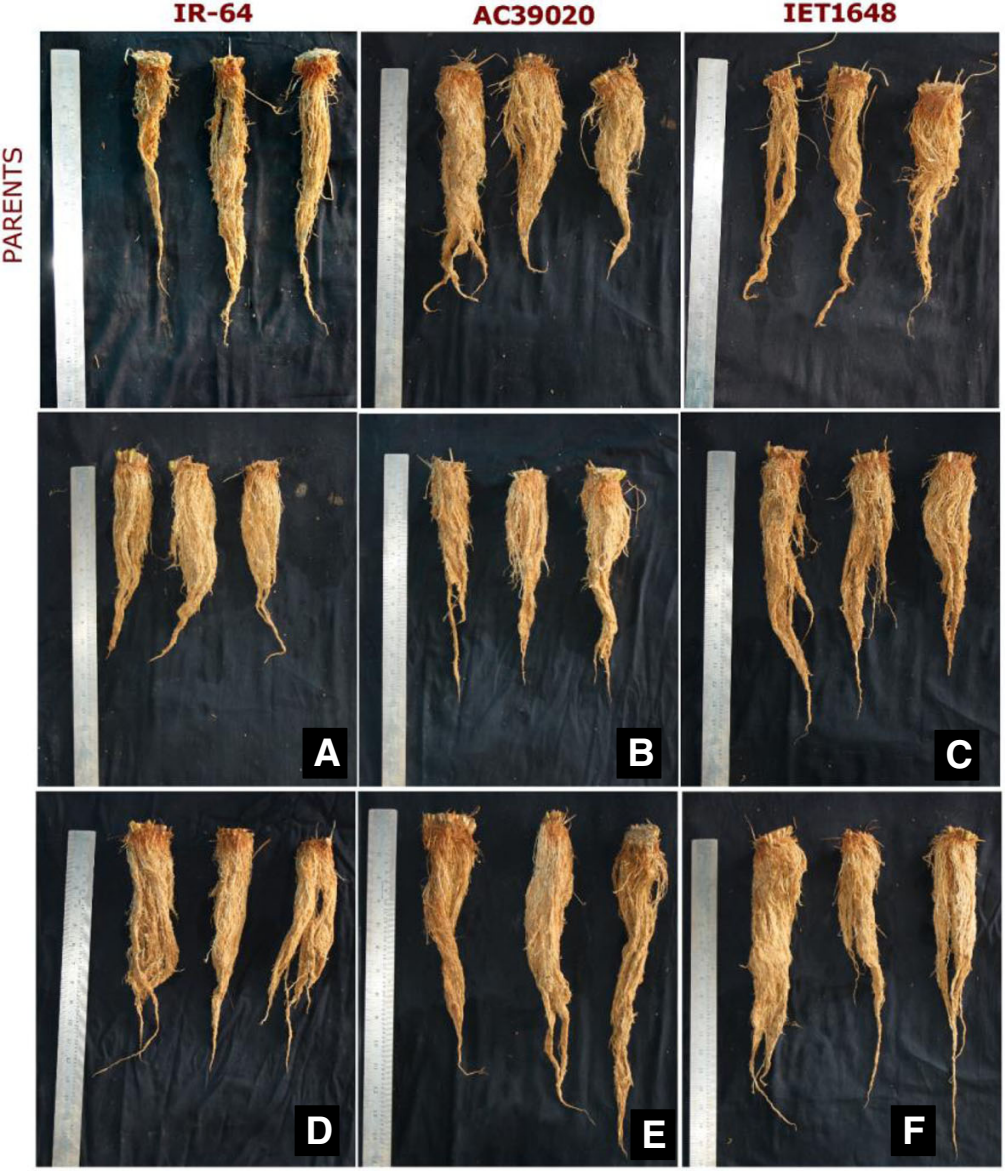
Improvement in root traits of TILs as compared to parents. **a** -TIL-32-1-40-84-54, **b** -32-1-40-24-57, **c** -32-1-40-78-84, **d** -81-23-27-378-93, **e** -81-23-31-75-157, **f** -81-23-31-75-187 Foot note: Plants were raised in root structure and the roots were harvested at 75DAS. The panel **a** to **f** represent the improved root traits in TILs

The average root length and root weight of the selected 260 TILs was 33.8 cm and 4.9 g pl^− 1^, respectively and the corresponding values for IR-64 were 26 cm and 3.2 g pl^− 1^. A good number of TILs showed higher root length and root weight than IR-64 and the root donor genotype AC-39020 (Table 5, Fig. 2 and Additional file 1: Figure S5). Similarly, the average Δ^13^C of the selected TILs was 19.57 which was significantly lower than that for IR-64 (Table 5 and Additional file 1: Figure S5) A significantly higher TDM of the selected 260 TILs compared with IR-64 emphasized the relevance of introgressing root and WUE traits.

**Table 5:** Variability in root traits and other biometric parameters among parents and BC_3_F_3_ progenies

Parents/Progenies	PH	RL	RW	SLA	TLA	Δ^13^C	TDM	DM/LA
IR-64	80	26	3.20	208	3467	22.42	45.40	152.3
AC-39020	105	41	7.40	216	4732	22.31	71.10	198.4
IET-16348	94	33	3.40	188	3533	19.43	56.20	165.3
260 lines	83	34	4.90	202	3234	19.57	59.60	176.4
Minimum	65	20	1.54	182	2342	16.31	40.00	80
Maximum	85	70	12.2	232	5343	21.56	120.00	220
CD	4.6	5.8	1.90	52.16	987	1.15	13.13	8.56
CV	5.4	12.3	8.60	13.5	14.5	5.40	7.60	16.6

Note: *PH* Plant height (cm), *RL* Root length (cm), *RW* Root weight (g pl^−1^), *TLA* Total leaf area (cm^2^ pl^−1^), *SLA* Specific leaf area (cm^2^ g^−1^), *Δ*^*13*^*C* Carbon isotope discrimination (‰), *TDM* Total dry matter (g pl^− 1^), *DM/LA* Dry matter per unit leaf area (g m^−2^)

In semi-irrigated aerobic condition, there would be more water in deeper soil profiles and hence extraction through better roots system would be useful. However, the imminent threat of exhausting soil moisture looms with deep rootedness (Farooq et al. 2009). Therefore, improving WUE becomes essential. Despite the theoretical importance of WUE in determining crop growth rates (Passioura, 1986), there has not been any great enthusiasm among the breeders to exploit the observed genetic variability for crop improvement. Evolutionarily plant maximized WUE predominantly through a reduction in transpiration. Though this water conservation is a useful strategy for survival, reduced transpiration is also associated with a concomitant reduction in CO_2_ influx for photosynthesis. Therefore, selecting for high WUE often resulted in reduced growth rates (Richards et al. 2002; Blum 2005, Sheshshayee et al. 2003; Condon et al. 2004). On the other hand, it is argued that an effective use of water is more relevant for improving growth rates (Blum 2005). We suggest that an effective use of water is possible when plants extract more water from deeper profiles of soil and uses the water more efficiently for biomass production. When water use is combined with WUE, unproductive water loss can be minimized hence improving water productivity. Therefore, it’s quite perceivable that a genotype that possesses high WUE despite deep rootedness will have superior growth rates and biomass accumulation capacity. The 260 TILs with better root and WUE indeed showed significantly higher biomass and net assimilation rate (DM/LA) (Table 5).

### Experiment 2- Productivity of BC_3_F_3_ Lines Under Semi-Irrigated Aerobic Condition

IR-64 is a widely adapted high yielding mega variety cultivated predominantly under puddle conditions is a drought sensitive cultivar (Serraj et al. 2011). It is well documented that the reduction in yield of IR-64 under water limited conditions is primarily due to increased spikelet sterility, especially when stress occurs during reproductive stage of crop growth. The shallow rootedness of IR-64 is often attributed to reduced water relations of both leaf and spikelets (Kamoshita et al. 2008).

In the present field experiment also IR-64 recorded a mean spikelet fertility of 54%, while the deep rooted donor genotype was significantly higher with 83% spikelet fertility (Table 6). Average yield of the 260 promising TILs at BC_3_F_3_ generation was significantly higher (27 g pl^− 1^) than IR-64 (22 g pl^− 1^) by over 17%. Though the minimum yield value of some of the TILs was as low as 7.5 g pl^− 1^, 75% of the TILs recorded more yield than IR-64. This segregation ratio of 1:3 indicates the selection of dominant characters that improved yield among most of the TILs. All the selected TILs had lower Δ^13^C (16 to 21.6 ‰) than IR-64 (22.4 ‰) and 83% of all the TILs recorded higher root biomass than IR-64. The spikelet fertility of TILs ranged between 65 and 95% which was significantly more than that of IR-64 (Table 6).

**Table 6:** Variability in yield and yield-related traits among parents and BC_3_F_3_ progenies for under semi-irrigated aerobic condition

Parents/Progenies	DFF	PN	# of S/P	TW	PL	SF	GY	GL	GW
IR-64	84	22	123	24	18	54	23	9.10	2.10
AC-39020	103	11	82	34	17	83	23	6.00	3.00
IET-16348	95	21	159	27	17	72	26	7.60	2.20
260 lines	84	23	117	27	18	80	32	8.88	2.06
Minimum	76	18	97	21	10	65	8	7.60	1.28
Maximum	95	28	143	29	21	95	50	9.77	2.41
CD	6.4	4.2	7.6	3.3	3.9	12.4	6.12	0.522	0.32
CV	3.6	2.1	4.3	6.8	5.4	5.5	7.6	5.6	4.3

Note: *DFF* Days to 50% flowering (days), *PN* Panicle number (#), *# of S/P* Number of spikelets per panicle (#), *TW* Test weight (g), *PL* Panicle length (cm), *SF* Spikelet fertility (%), *GY* Grain yield (g pl^−1^), *GL* Grain length (mm), *GW* Grain width (mm)

### Comparative Stress Response of TILs Under Managed Drought Environment

Aerobic cultivation conditions are characterized by periodic water stress (between irrigation schedules) and a higher VPD during the entire growing season. Under the conditions that favor transpiration, water productivity can only be enhanced when WUE is also increased. Five TILs with high root, low Δ^13^C and similar leaf area were selected from BC_3_F_3_ to ascertain the field level performance under managed drought environment (MDE) condition. This MDE facility with a mobile rain-out shelter effectively mimics aerobic field conditions by maintaining specific soil moisture status throughout the crop growth period. The selected five TILs (BC_3_F_4_ lines) were grown in plots of 2 m^2^ size under two moisture regimes namely, 100% FC and 60% FC.

Aerobic cultivation of rice is characterized by surface irrigation once in 5–6 days. In this practice, the soil is never saturated and there is a possibility of water percolating into deeper layers. Thus, the soil progressively dries and an intermittent water limited situation is often created between the episodes of irrigation (Bouman and Tuong 2001) Therefore, the two soil moisture regimes selected in the MDE study represent the entire range of condition that is experienced under aerobic conditions.

**Fig. 3:**
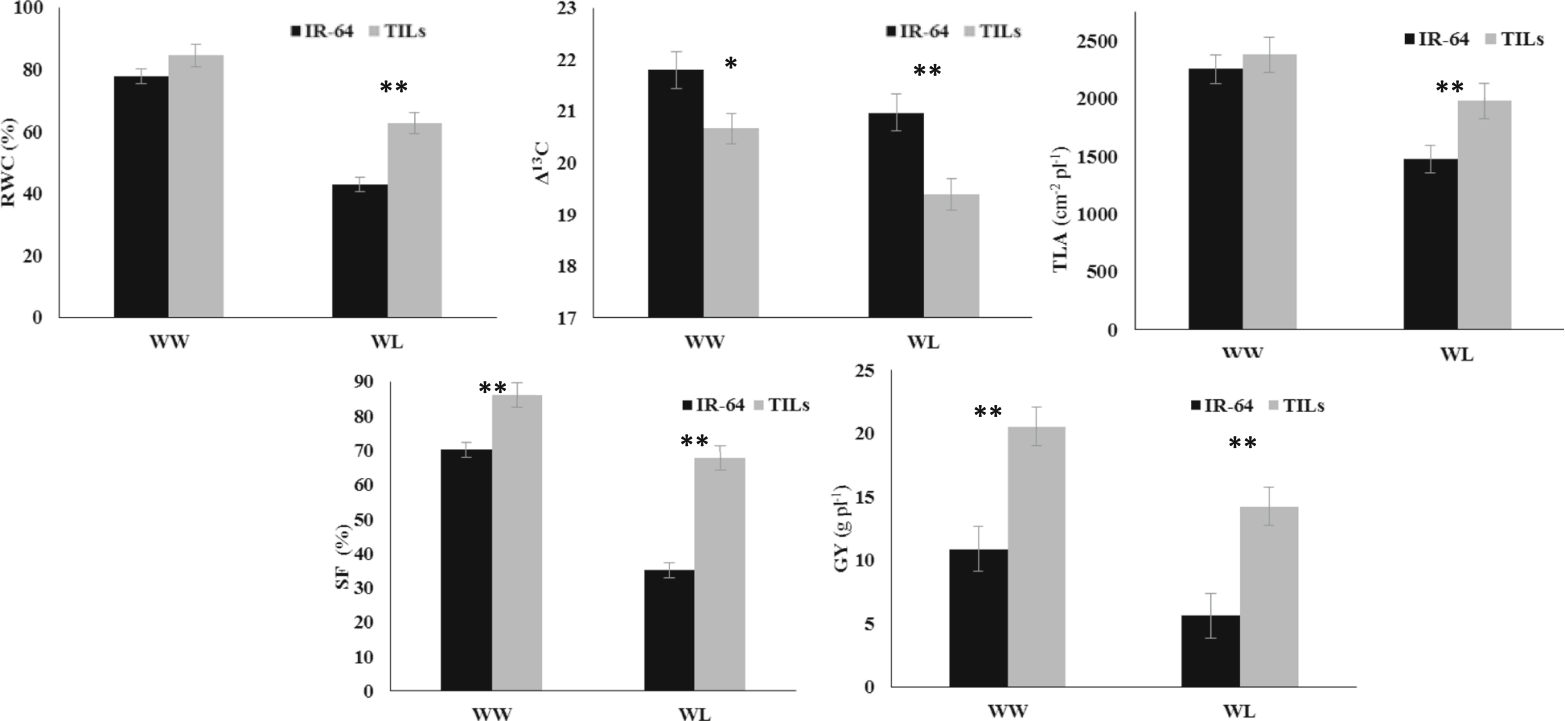
Comparison of selected trait introgressed lines with IR-64 for RWC, Δ^13^C, TLA, spikelet fertility and grain yield under managed drought environment. *, ** indicates a significant difference at *P* =0.05, 0.01. Note: Selected five TILs along with the IR-64 were grown under well watered (100% FC) and water limited (60% FC) conditions under MDE. Various physiological parameters were measured at vegetative and reproductive stage and compared with IR-64

Under this condition, we tested the yield performance of the five selected TILs in comparison with IR-64. The mean yield of five TILs under both water regimes was 52% (100% FC) and 63% (60% FC) higher than IR-64. The RWC of IR-64 and the five TILs was comparable under 100% FC treatment. Though both IR-64 and the TILs had reduced RWC under water limited condition, the TILs maintained a significantly higher tissue water status than IR-64 (Fig. 3). The canopies of TILs were significantly cooler compared to IR-64, especially under water limited condition (data not given). Stomatal closure is one of the fastest responses under water limited condition. This often leads to decrease in intercellular CO_2_ concentration (Ci) and hence a decreased Δ^13^C is often noticed (Hubick et al. 1988). TILs on an average revealed lower Δ^13^C than IR-64 in both 100% FC and 60% FC conditions. Besides stomatal conductance (g_s_), the carboxylation capacity can also alter Ci levels and hence Δ^13^C. The TILs are characterized by a higher carbon assimilation capacity and hence a lower Δ^13^C under any condition is expected.

Introgression of root and WUE rendered the plants to maintain tissue water relations as well as metabolism under decreased water status. Thus, the spikelet fertility of TILs was significantly higher even under water limited condition and hence had higher grain yield (Fig. 3).

**Fig. 4:**
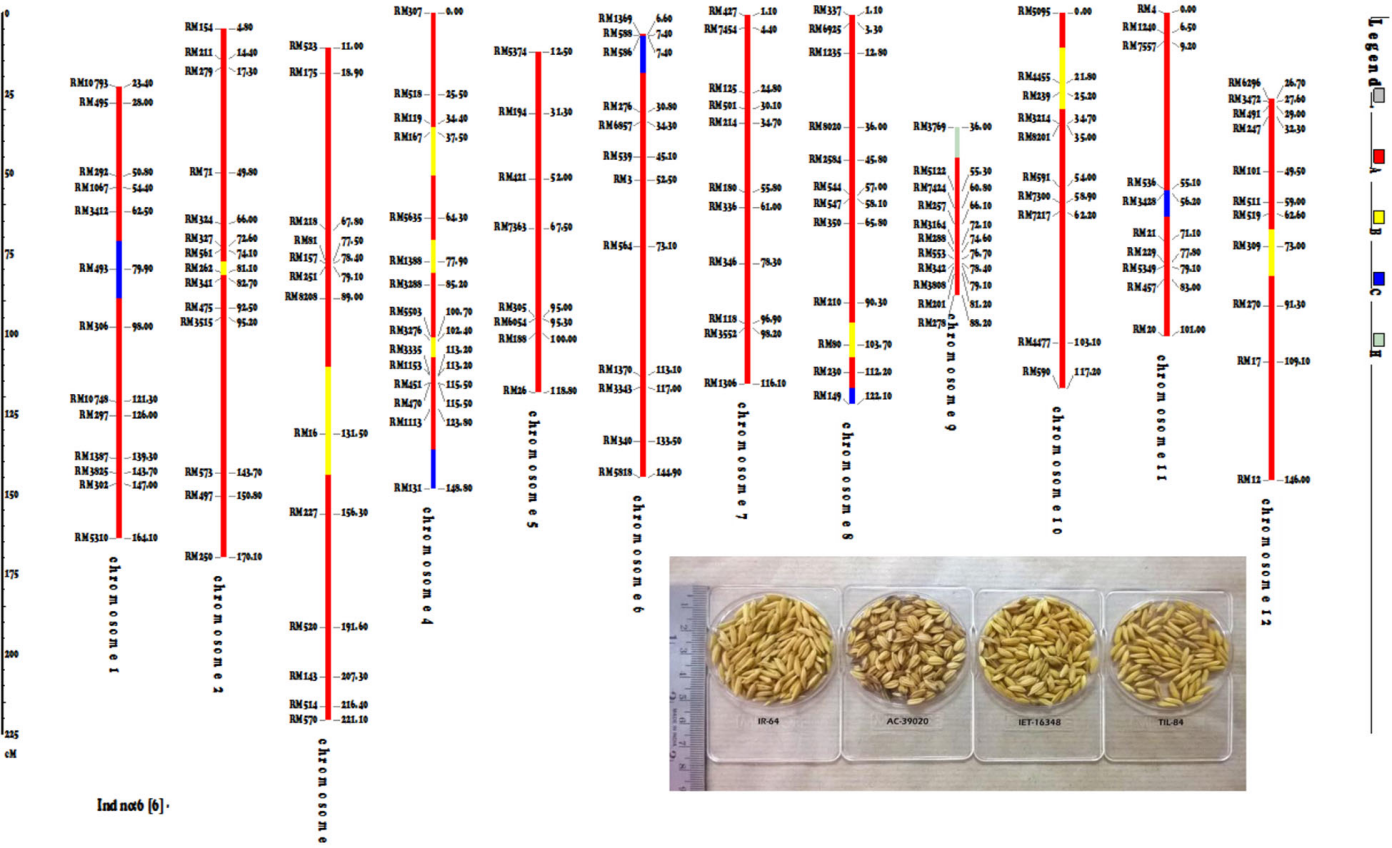
Graphical genotyping plot and the grain characters (inset) for a selected TIL namely 32–1–40-78-84. Note: Red colour indicates homozygous region similar to IR-64 genome, Yellow colour indicate introgression of AC-39020 genome governing root QTLs and Blue colour indicates introgression of IET-16348 genome for WUE traits

The morphological characters of TILs were highly comparable with IR-64, indicating the recovery of IR-64 characters. As an evidence of the recurrent parent genome recovery, graphical genotyping and grain characteristics of one TIL viz. 32–1–40-78-84 is given in Fig. 4. The detailed information about the genome recovery from BC_2_F_1_ till BC_3_F_4_ for the selected best TILs is given in Additional file 4: Table S5.

The genome integration data along with these morphological similarities with IR-64 emphasizes the recovery of recurrent parent genome with the integration of desirable target traits (Additional file 1: Figure S6). Some of the morpho-physiological characters such as leaf size and grain characters for the parents and the TILs are given in Additional file 1: Table S6.

These results clearly illustrate that introgressing diverse physiological and morphological traits alone can significantly improve productivity under water limited conditions, while saving irrigation water. This work provides a proof of concept that improving physiological traits associated with maintenance of tissue turgor and metabolism is the most appropriate strategy to enhance growth rates and productivity under water limited conditions.

## Conclusion

We provided experimental evidences to prove that introgression of root traits to harness water from deeper soil layers and efficient use of water for biomass production, can substantially increase yield levels under water limiting semi-irrigated aerobic conditions. A novel approach of using multiple trait donor genotypes and selecting promising lines using SSR markers associated with WUE and root traits accelerated trait introgression.. Improvements in root traits and WUE resulted in higher spikelet fertility among the TILs and carbon assimilatory capacity. This resulted in the trait introgressed lines showing more than 30% increase in productivity under water limiting conditions of aerobic cultivation. With a significantly high level of genome recovery, the TILs can be considered as “improved IR-64” with enhanced productivity and drought adaptability. To our knowledge, this is the first successful report of breeding to improve physiological traits in rice.

## Additional files

Additional file 1: **Figure S1.** Root phenotyping using root structures. **Figure S2.** Identification of trait introgressed lines for advancing at BC_3_F_2_ stage. **Figure S3.** Frequency distribution of BC_3_F_2_ population for various morpho-physiological traits under aerobic condition. # indicates the value for recurrent parent IR-64. **Figure S5.** Frequency distribution of BC_3_F_3_ population for various morpho-physiological traits in root structure. **Figure S6.** Improved phenotype of the selected TILs along with parents. **Table S1.** Background markers used in the study for reconstructing IR-64 background. **Table S2.** Differences in weather parameters between experimental locations (Bengaluru and Mandya). **Table S3.** Improvement of yield and yield-attributes among selected trait introgressed BC_3_F_1_ progenies. **Table S6.** Morphophysiological characters of TILs under well-watered and water limited condition along with IR-64. (DOCX 4617 kb)

Additional file 2: **Table S4.** Foreground genotyping of trait introgressed lines using associated markers for specific target traits. (XLSX 24 kb)

Additional file 3: **Figure S4.** Representative gel images derived from MultiNA for segregating BC_3_F_2_ progenies (A) and trait introgressed BC_3_F_3_ progenies (B) along with parents. (DOCX 365 kb)

Additional file 4: **Table S5.** Genotyping of the selected TILs with SSR markers for Background genome recovery and their chromosomal positions. (XLSX 18 kb)

## Abbreviations

BGS: Background section
DM/LA: Dry matter per unit leaf area
FC: Field capacity
FGS: Foreground selection
MABC: Marker assisted backcross
MDE: Managed drought environment
NAR: Net assimilation rate
QTL: Quantitative trait loci
RWC: Relative water content
SLA: Specific leaf area
TILs: Trait introgressed lines
WUE: Water use efficiency
