# Supplementary material for: Introgression of Root and Water Use Efficiency Traits Enhances Water Productivity: An Evidence for Physiological Breeding in Rice (Oryza sativa L.)
Source: Rice (N Y). 2019 Mar 7;12:14. doi: 10.1186/s12284-019-0268-z (PMC6405788; doi:10.1186/s12284-019-0268-z)
Supplement: Supplementary file 1 — Figure S1. Root phenotyping using root structures. Figure S2. Identification of trait introgressed lines for advancing at BC3F2 stage. Figure S3. Frequency distribution of BC3F2 population for various morpho-physiological traits under aerobic condition. # indicates the value for recurrent parent IR-64. Figure S5. Frequency distribution of BC3F3 population for various morpho-physiological traits in root structure. Figure S6. Improved phenotype of the selected TILs along with parents. Table S1. Background markers used in the study for reconstructing IR-64 background. Table S2. Differences in weather parameters between experimental locations (Bengaluru and Mandya). Table S3. Improvement of yield and yield-attributes among selected trait introgressed BC3F1 progenies. Table S6. Morphophysiological characters of TILs under well-watered and water limited condition along with IR-64. (DOCX 4617 kb) [file 12284_2019_268_MOESM1_ESM.docx]

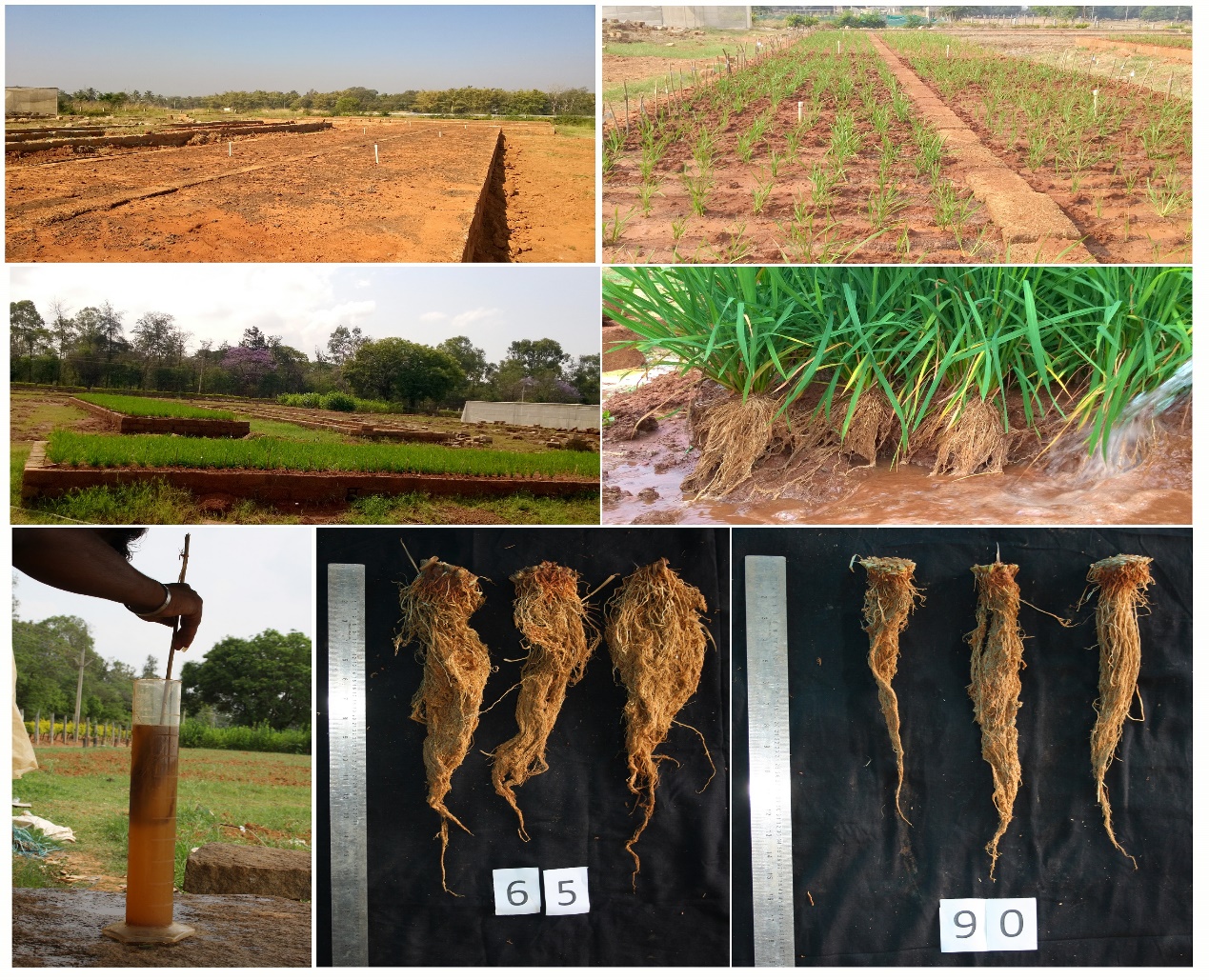


**A**

**B**

**C**

**D**

**E**

**F**

**Additional file 1**

**Figure S1.**  Root phenotyping using root structures.

Foot note: Specially designed root structure (Raju *et al.,* 2014) ensures accurate phenotyping for root traits under natural conditions and with interplant competition like in real field conditions (A-C). The soil can be washed by dismantling the side walls using a jet water (D). Determination of root volume by water displacement approach (E). Genetic variability in root traits (F).

**Figure S2.** Identification of trait introgressed lines for advancing at BC_3_F_2_ stage.


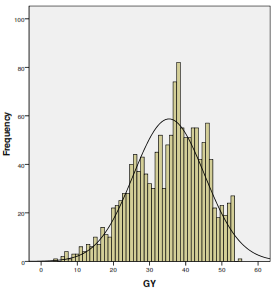


**#**


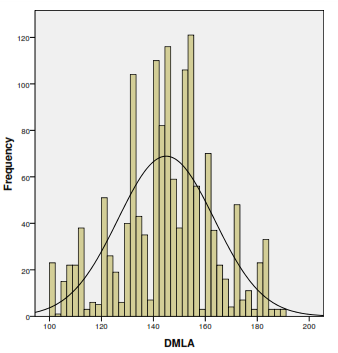


**#**


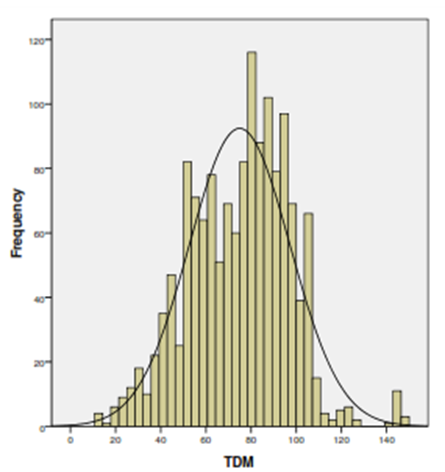


**#**


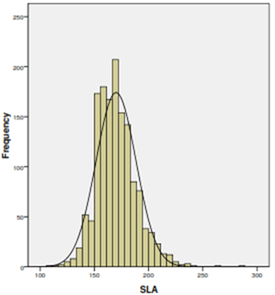


**#**

**Figure S3.** Frequency distribution of BC_3_F_2_ population for various morpho-physiological traits under aerobic condition. # indicates the value for recurrent parent IR-64.

Foot note: SLA – Specific leaf area (cm^2^ g^-1^), TDM – Total dry matter (g pl^-1^), DM/LA – Dry matter per unit leaf area (g mt^2^), GY – Grain yield (g pl^-1^).


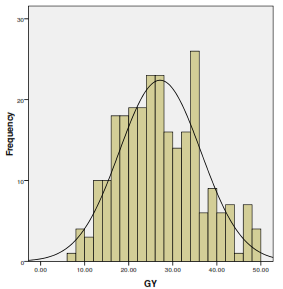


**#**


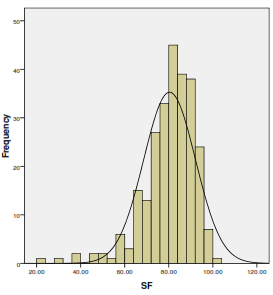


**#**


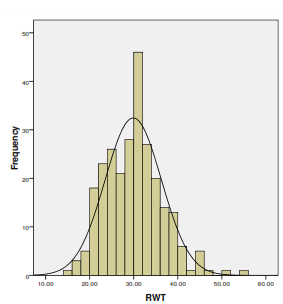


**#**


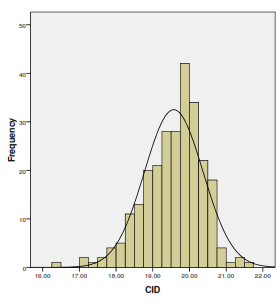


**#**


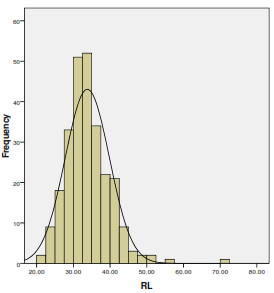


**#**


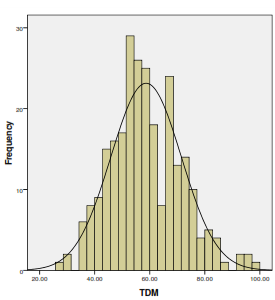


**#**

**Figure S5.**  Frequency distribution of BC_3_F_3_ population for various morpho-physiological traits in root structure. # indicates the value for recurrent parent IR-64.

Foot note: RW - Root weight (g pl^-1^), RL – Root length (cm), Δ^13^C - Carbon isotope discrimination (parts per mill), TDM - Total dry matter (g pl^-1^), GY – Grain yield (g pl^-1^), SF – Spikelet fertility (%).

**Figure S6.** Improved phenotype of the selected TILs along with parents. A - IR-64, B - AC-39020, C - IET-16348,D - TIL-32-1-40-84-54, E - 32-1-40-24-57, F - 32-1-40-78-84, G - 81-23-27-378-93, H - 81-23-31-75-157


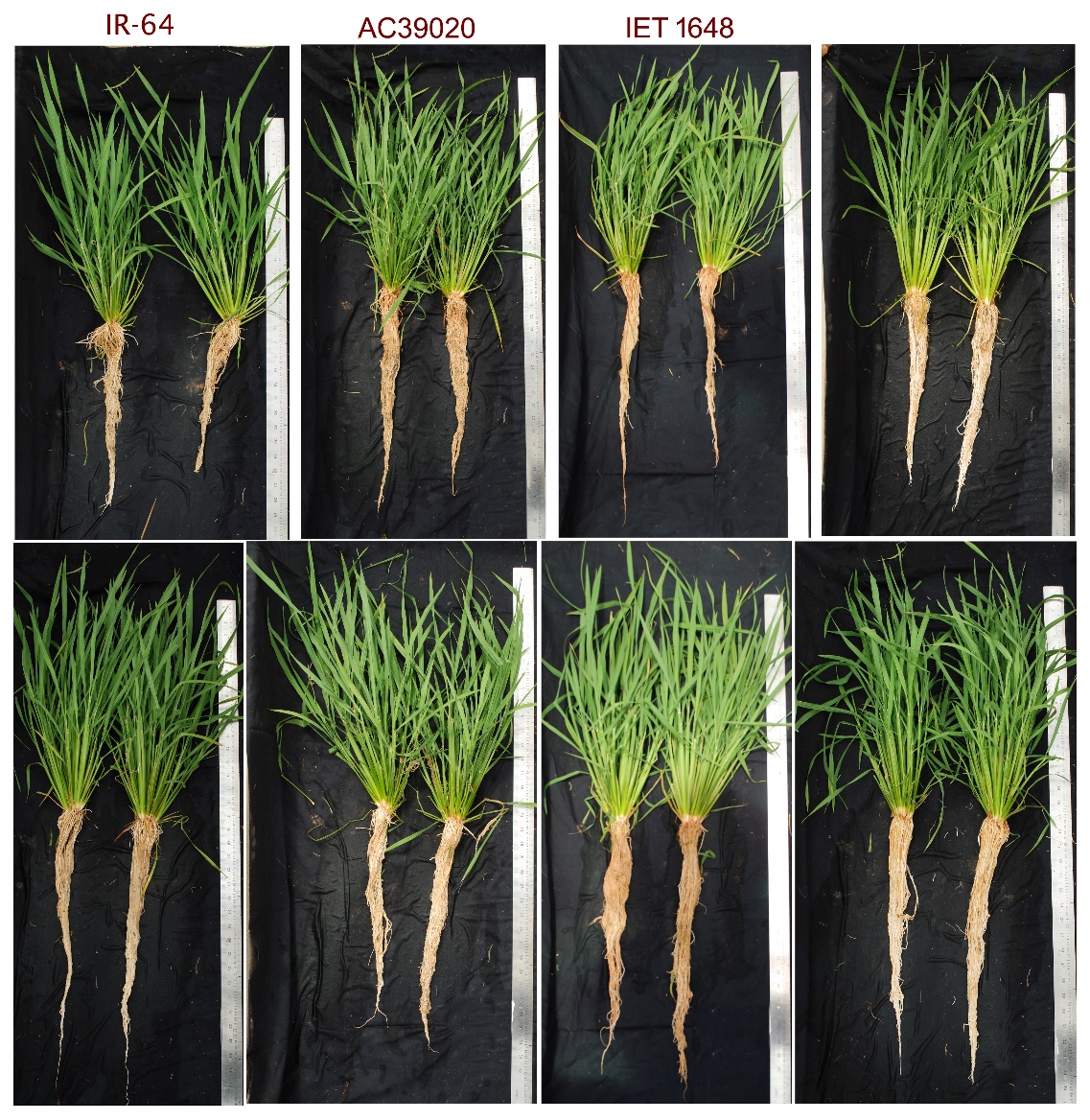


**A**

**B**

**C**

**D**

**E**

**F**

**G**

**H**

**Table S1.** Background markers used in the study for reconstructing IR-64 background

| **Chr. #** | **No. of markers analysed** | **Total number of polymorphic markers** | **Name of the polymorphic markers** |
| --- | --- | --- | --- |
| 1 | 38 | 11 | RM10793, RM495, RM292, RM1067, RM3412, RM306, RM10748, RM297, RM1387, RM302, RM5310 |
| 2 | 34 | 12 | RM154, RM211, RM279, RM324, RM327, RM561, RM341, RM475, RM3515, RM573, RM497, RM250 |
| 3 | 37 | 13 | RM523, RM175, RM218, RM81, RM157, RM251, RM8208, RM16, RM227, RM520, RM143, RM514,RM570 |
| 4 | 33 | 15 | RM307, RM518, RM119, RM167, RM5635, RM1388, RM3288, RM5503, RM3276, RM3335, RM1153, RM470, RM451, RM1113, RM131 |
| 5 | 37 | 8 | RM5374, RM194, RM421, RM7363, RM305, RM6054, RM188, RM26 |
| 6 | 37 | 11 | RM1369, RM588, RM276, RM6857, RM539, RM3, RM564, RM1370, RM3343, RM340, RM5818 |
| 7 | 42 | 11 | RM427, RM7454, RM125, RM501, RM214, RM180, RM336, RM346, RM118, RM3552, RM1306 |
| 8 | 36 | 9 | RM337, RM6925, RM1235, RM8020, RM544, RM547, RM350, RM210, RM230 |
| 9 | 32 | 11 | RM3769, RM5122, RM7424, RM257, RM3164, RM288, RM553, RM342, RM3808, RM201, RM278 |
| 10 | 41 | 8 | RM5095, RM3214, RM8201, RM591, RM7300, RM7217, RM4477, RM590 |
| 11 | 39 | 10 | RM4, RM1240, RM7557, RM536, RM3428, RM21, RM229, RM5349, RM457, RM20 |
| 12 | 36 | 10 | RM6296, RM3472, RM491, RM101, RM511, RM519, RM309, RM270, RM17, RM12 |

**Table S2.** Differences in weather parameters between experimental locations (Bengaluru and Mandya)

| **Experiment** | **Root traits and WUE** | **Yield parameters** |
| --- | --- | --- |
| Place | GKVK campus, Bengaluru | VC Farm, ZARS, Mandya |
| Year and season | 2015 (Summer) | 2015 (Summer) |
| Latitude and longitude | 13° 05'N;770 34'E | 12°.31' 27'N;76.530 44'E |
| Altitude (MSL) | 930 | 678 |
| Mean sun shine hours | 8.6 | 8.8 |
| Mean temperatures (^o^ C)(mini-max) | 22.3-33.6 | 25.3-34.6 |
| Mean relative humidity (%) | 60.8 | 65.8 |
| Average annual rainfall (mm) | 859 | 700 |
| Site/conditions | Aerobic condition | Aerobic and puddled |
| Soil type | Red loamy | Red loamy and clay loamy |

**Table S3.** Improvement of yield and yield-attributes among selected trait introgressed BC_3_F_1_ progenies

| **Trait** | **IR-64** | **Selected plants in DCBC_3_F_1_** | | | |
| --- | --- | --- | --- | --- | --- |
|  |  | **32-1-34** | **32-1-40** | **81-23-107** | **81-23-115** |
| SLA | 188.04 | 157.24 | 179.54 | 136.8 | 226.5 |
| TDM | 62.75 | 118.50 | 119.4 | 117.4 | 122.1 |
| GY | 28.50 | 57.00 | 58.40 | 47.40 | 48.10 |
| HI | 0.45 | 0.48 | 0.49 | 0.40 | 0.39 |
| SF | 60.69 | 85.57 | 86.36 | 90.24 | 75.77 |
| GL/GW | 4.57 | 4.27 | 4.08 | 3.78 | 4.53 |
| DM/LA | 135.6 | 167.6 | 156.6 | 165.4 | 134.4 |
|  |  |  |  |  |  |

Note: SLA – Specific leaf area (cm^2^ g^-1^), TDM – Total dry matter (g pl^-1^), GY – Grain yield (g pl^-1^), HI – Harvest index, SF – Spikelet fertility (%), GL/GW – Grain length to width ratio, DM/LA – Dry matter per unit leaf area (g mt^2^).

Note: See Table 2 B for Δ^13^C and LT for these four trait introgressed BC_3_F_1_ plants

**Table S6:** Morphophysiological characters of TILs under well-watered and water limited condition along with IR-64

| **TIL #** | **PH** | | **SCMR** | | **TN** | | **PN** | | **TW** | | **GL/GW** | |
| --- | --- | --- | --- | --- | --- | --- | --- | --- | --- | --- | --- | --- |
|  | **WW** | **WL** | **WW** | **WL** | **WW** | **WL** | **WW** | **WL** | **WW** | **WL** | **WW** | **WL** |
| 32-1-40-84-54 | 63.00 | 56.83 | 38.00 | 32.77 | 34.00 | 29.67 | 32.33 | 19.33 | 27.18 | 18.17 | 2.6 | 2.1 |
| 32-1-40-24-57 | 70.67 | 63.56 | 36.13 | 34.80 | 31.67 | 28.00 | 22.00 | 23.00 | 25.57 | 18.25 | 2.3 | 2.2 |
| 32-1-40-78-84 | 62.33 | 59.94 | 41.00 | 38.10 | 33.00 | 27.00 | 20.67 | 18.50 | 24.61 | 20.15 | 2.2 | 2.0 |
| 81-23-27-378-93 | 65.00 | 58.44 | 41.53 | 35.20 | 29.33 | 20.50 | 20.33 | 18.50 | 28.35 | 18.35 | 2.4 | 2.3 |
| 81-23-31-75-157 | 67.33 | 56.44 | 34.50 | 38.15 | 28.00 | 26.00 | 20.50 | 18.00 | 25.98 | 17.63 | 2.5 | 2.2 |
| IR-64 | 55.33 | 50.78 | 30.93 | 32.75 | 25.33 | 13.33 | 18.00 | 15.00 | 24.30 | 16.10 | 2.2 | 2.0 |
| CD | 5.8 | | 3.6 | | 5.4 | | 4.5 | | 4.3 | | 0.01 | |
| CV | 8.4 | | 10.4 | | 10.5 | | 9.2 | | 3.4 | | 2.1 | |

Note: WW – Well watered (100% FC), WL – Water limited (60 % FC)

PH – Plant height (cm), SCMR – SPAD chlorophyll meter reading, TN – Tiller number (#), PN – Panicle number (#), TW – Test weight (g),

GL/GW – Grain length to width ratio.
