# Supplementary material for: Introgression of Root and Water Use Efficiency Traits Enhances Water Productivity: An Evidence for Physiological Breeding in Rice (Oryza sativa L.)
Source: Rice (N Y). 2019 Mar 7;12:14. doi: 10.1186/s12284-019-0268-z (PMC6405788; doi:10.1186/s12284-019-0268-z)
Supplement: Supplementary file 3 — Figure S4. Representative gel images derived from MultiNA for segregating BC3F2 progenies (A) and trait introgressed BC3F3 progenies (B) along with parents. (DOCX 365 kb) [file 12284_2019_268_MOESM3_ESM.docx]

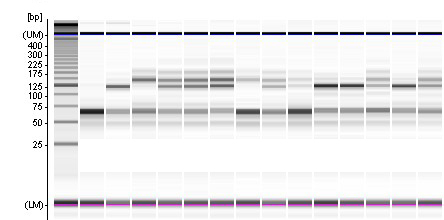


**L P_1_ P_2_ P_3_  1 2 3 4 5 6 7 8 9 10 11**


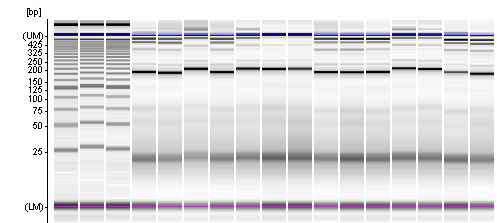


**L_1_ L_2_ L_3_ P_1_ P_2_ P_3_  1 2 3 4 5 6 7 8 9 10 11**

**Additional file 3: Figure S4** Representative gel images derived from MultiNA for segregating BC_3_F_2_ progenies (A) and trait introgressed BC_3_F_3_ progenies (B) along with parents.
